# Calcium Dynamics Mediated by the Endoplasmic/Sarcoplasmic Reticulum and Related Diseases

**DOI:** 10.3390/ijms18051024

**Published:** 2017-05-10

**Authors:** Florence N. Reddish, Cassandra L. Miller, Rakshya Gorkhali, Jenny J. Yang

**Affiliations:** Department of Chemistry, Center for Diagnostics and Therapeutics (CDT), Georgia State University, Atlanta, GA 30303, USA; freddish1@student.gsu.edu (F.N.R.); cmiller88@student.gsu.edu (C.L.M.); rgorkhali1@student.gsu.edu (R.G.)

**Keywords:** calcium signaling, endoplasmic reticulum, sarcoplasmic reticulum, RyR, IP_3_R, SERCA pump, JP45, calsequestrin, GECI

## Abstract

The flow of intracellular calcium (Ca^2+^) is critical for the activation and regulation of important biological events that are required in living organisms. As the major Ca^2+^ repositories inside the cell, the endoplasmic reticulum (ER) and the sarcoplasmic reticulum (SR) of muscle cells are central in maintaining and amplifying the intracellular Ca^2+^ signal. The morphology of these organelles, along with the distribution of key calcium-binding proteins (CaBPs), regulatory proteins, pumps, and receptors fundamentally impact the local and global differences in Ca^2+^ release kinetics. In this review, we will discuss the structural and morphological differences between the ER and SR and how they influence localized Ca^2+^ release, related diseases, and the need for targeted genetically encoded calcium indicators (GECIs) to study these events.

## 1. Introduction

From transcription [1] to cell growth and proliferation [2], intracellular calcium (Ca^2+^) transients activate various internal processes that sustain the life of an organism. The efficiency and speed of Ca^2+^ signaling is powered by the ability of cells to maintain the near 20,000-fold gradient between intracellular and extracellular Ca^2+^ concentrations [3]. Resting cytosolic Ca^2+^ concentration in non-excitable cells is about 0.1 µM. When cells are activated, the level of cytosolic Ca^2+^ rises up to 1 µM, triggering many consequent processes [4]. In skeletal muscle cells, cytosolic Ca^2+^ can far exceed 1 µM during activation [5]. This rise in cytosolic Ca^2+^ can control a vast repertoire of cellular functions because of the adaptable nature and organization of the Ca^2+^ signaling system. The Ca^2+^ channels, pumps, and calcium-binding proteins (CaBPs) expressed in different tissues, i.e., the Ca^2+^ signaling toolkit, are tailored to yield Ca^2+^ signaling mechanisms that produce varied spatial-temporal patterns for the initiation of slow processes such as gene expression [6] to fast processes such as neurotransmitter release [7,8]. The large pool of intracellular Ca^2+^ that initiates these subsequent processes comes from the intracellular Ca^2+^ storage organelles, primarily the endoplasmic reticulum (ER) and its specialized form, the sarcoplasmic reticulum (SR), in muscle cells.

The ER is a dynamic organelle that is purposed with vital tasks within the cell such as protein synthesis and folding and intracellular signaling [9]. The ER reacts to intracellular cues from inositol 1,4,5-trisphosphate (IP_3_), Ca^2+^, and reactive oxygen species (ROS), to name a few, by transmitting a Ca^2+^ signal or stress signal to recruit subsequent components to produce a global effect. The ER is central to intracellular Ca^2+^ signaling. The inositol 1,4,5-trisphosphate receptor (IP_3_R) and the ryanodine receptor (RyR) are the primary Ca^2+^ release channels situated on the ER and SR membrane [10,11]. Calcium activation of these receptors creates a regenerative process of calcium release called calcium induced calcium release (CICR), which contributes largely to rapid intracellular Ca^2+^ transients. CICR has been studied extensively in muscle cells and neurons [8,12,13,14] including cardiac neurons [15]. Ligands such as IP_3_ and cyclic ADP-ribose (cADPR) are known to sensitize IP_3_R and RyR to calcium, respectively, where calcium alone can elicit calcium release through the RyR [16,17]. The sarco-endoplasmic reticulum Ca^2+^ ATPase (SERCA) pump is located on the ER/SR membrane and functions to restore resting cytosolic [Ca^2+^] by refilling the ER/SR with Ca^2+^ upon signal termination [18]. Intraluminal ER Ca^2+^ also mediates the function of the ER [19]. Fluctuations in ER Ca^2+^ impact protein folding and can trigger the unfolded protein response (UPR) [20].

The heterogeneity and plasticity of the ER allows for the modulation of its function [21]. The ER exists as a membranous network of sheets and tubules [22]. Due to its vast network, the ER forms connections with other membrane systems through membrane contact sites (MCS) in the cell. These associations are formed with organelles such as the Golgi and mitochondria and with larger bilayers such as the plasma membrane (PM). Transport of proteins from the ER to the Golgi is the first step in the secretory pathway. The sorting of these proteins occurs at the ER–Golgi interface. These contacts are primarily formed between areas of flattened ER and the Golgi cisternae [23,24]. In the mitochondria, tubule regions of the ER associate with the organelle at the mitochondria-associated ER membrane (MAM). These regions are linked to such cellular processes as metabolism, lipid synthesis, cell death, and Ca^2+^ signaling [25]. Outer ER membranes form adjacent contacts with the PM. ER–PM junctions vary in their inherent features depending on the cell type [26,27,28,29]. The uniqueness of these junctions highlight their role in exocytosis, endocytosis, lipid homeostasis, and Ca^2+^ signaling [30]. The distribution of the ER/SR in the cell and associated Ca^2+^ signaling proteins control the spatial signaling ability of the organelle [23]. This review will discuss the morphology and structural organization of the ER/SR and how it contributes to the local differences in Ca^2+^ release, the role of the ER/SR in disease, and the development of targeted genetically encoded calcium indicators (GECIs) to study ER/SR-mediated Ca^2+^ signaling, especially related to rapid calcium transients.

## 2. Morphology of the ER and the SR

**Figure 1:**
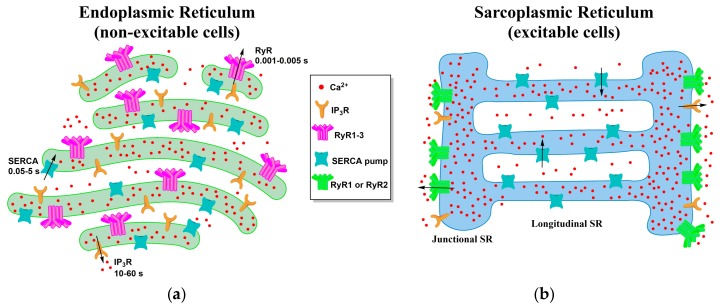
A comparison of the ER and SR morphology and the arrangement of the common surface receptors and pumps. (**a**) The ER, found in all cell types, excitable and non-excitable but mainly non-excitable cells, have an even distribution of the receptors that release Ca^2+^ from the organelle, IP_3_R and RyR, and the pump which refills this organelle, the SERCA pump. Notably, the isoforms of the receptors and pumps expressed hinge upon the tissue they are located; (**b**) The SR, found only in excitable cells, has a higher distribution of RyR, primarily concentrated in the TC, and the SERCA pump being on the longitudinal SR. Additionally, the RyR isoforms 1 and 2 are more predominately found in the SR than isoform 3. The black arrows indicate the direction Ca^2+^ flows through the respective receptor or pump.

The ER appears as an uninterrupted network with three distinct forms and accompanied specialized functions [22] (Figure 1a). The rough ER (RER) appears as flattened sacs and is speckled with ribosomes for protein synthesis. The smooth ER (SER) is an elongated, cylindrical network with key functions in Ca^2+^ storage and release. Surrounding the nucleus is an extension of the ER called the nuclear envelope (NE). The NE is also dotted with ribosomes and supplies the nucleus with Ca^2+^ for gene transcription [22,31]. The ER extends throughout the cell taking up more than 10% of the cell capacity. As such the ER is able to interact with several key organelles and membranes, namely the plasma membrane, mitochondria, lysosomes, and endosomes. The local regulation of these microdomain interactions with the ER allow for comprehensive control of cellular Ca^2+^ dynamics [31,32].

The SR is a morphologically distinct version of the smooth ER specialized for Ca^2+^ release to fuel muscle contraction [33] (Figure 1b). It consists of large sink portions referred to as terminal cisternae (TC) connected to elongated tubes termed the longitudinal SR. RyRs are located solely in the TC, while SERCA pumps are found exclusively in the longitudinal SR. The distribution of these primary receptors within the SR divide this organelle into two divergent subdomains tasked with the release and the uptake of Ca^2+^ [34]. Within the skeletal muscle cell, the TC is distributed to be in proximity to the tubule invaginations of the sarcolemma membrane called the transverse tubules (t tubules) with triads consisting of a t tubule segment between two TC [35]. Located on the t tubule membrane are the dihydropyridine receptors (DHPRs) that allow Ca^2+^ to flow into the myoplasm in response to depolarization. The space between the SR TC membrane and the t tubule membrane is referred to as the junctional zone [36]. This extended SR network consisting of the TC, longitudinal SR, and the t tubules surrounds the myofibrils in repeating units near the sarcomere ensuring delivery of Ca^2+^ for contraction to occur [37].

## 3. ER/SR Mediated Calcium Signaling

The Ca^2+^ released from the ER/SR makes up the bulk of the Ca^2+^ signal. The IP_3_R and the RyR are the main Ca^2+^ release channels on the ER/SR membrane. For cardiac muscle or skeletal muscle, Ca^2+^ activates the RyR through CICR via interaction with the DHPR, allowing more Ca^2+^ to discharge from the ER/SR to propagate the Ca^2+^ signal [38,39,40]. The SERCA pump is the main mode of Ca^2+^ transport back from the cytosol into the ER/SR [41]. Here we will discuss the distribution and function of these calcium signaling constituents and their interaction with other proteins.

### 3.1. The Ryanodine Receptor

The RyR exists in three isoforms in mammals: RyR1, RyR2, and RyR3. RyR1 and RyR2 are the predominant isoforms found in skeletal and cardiac muscle, respectively, while RyR3 is dominant in the thalamus, hippocampus, corpus striatum, and smooth muscle [42,43]. RyR3 is also present in mammalian skeletal muscle cells during development [44]. High-resolution cryo-electron microscopy structures of the RyR show it exists as a homotetramer with a total mass over 2 MDa making it the largest ion channel. The large cytosolic portion of the RyR has a mushroom-like shape with the rest of the channel embedded in the SR membrane [45,46]. This large cytosolic portion of the RyR is where many proteins bind to regulate its function such as FKBP12 (RyR1), FKBP12.6 (RyR2), calmodulin (CaM), CaMKII, S100A proteins, and DHPRs [47]. Ca^2+^ activates and inhibits RyR1 activity. When Ca^2+^ concentration in the cytosol is ~1 µM, the receptor is active, and it is inhibited when the Ca^2+^ concentration reaches 1 mM. This activation occurs in a [Ca^2+^] dependent bell shaped curve [11]. Myoplasmic free magnesium (Mg^2+^) is a potent inhibitor of RyR1 in resting skeletal muscle cells. If the physiological concentration of magnesium is lowered from 1 to 0.05 mmol/L, the receptor opens resulting in a steep decrease in SR Ca^2+^. Cytosolic adenosine triphosphate (ATP) also triggers the Ca^2+^ release channel activity [48]. Caffeine exerts a stimulatory effect on RyRs by enhancing their affinity for Ca^2+^ without disrupting magnesium binding [49]. 4-Chloro-m-cresol (4-CmC) is a potent non-native agonist of the RyR, having a 10-fold higher sensitivity than caffeine for inducing SR Ca^2+^ release with both regularly employed to study RyR-mediated Ca^2+^ release in healthy and diseased cells [50]. While the three isoforms of RyR are predominately found in their specific cell types, they can be found in varying concentrations in all cells. Their location in the cell, specifically on the ER/SR membrane, varies between each isoform. RyR1 is highly localized in the terminal cisternae region of the SR membrane facing the t tubules in skeletal muscle cells. As mentioned previously, he DHPRs, situated on the t tubule membrane, interact directly with RyR1 to activate Ca^2+^ release when membrane depolarization occurs [36]. Residues 1635–2636 of RyR1 were shown to interact with the DHPR to mediate Ca^2+^ release and excitation–contraction (E-C) coupling [51]. Later, it was discovered that residues 1–1680 at the N-terminal portion of RyR1 facilitate E-C coupling [52]. RyR1 also enriches the activity of the DHPR through a backward current [53]. RyR1 and RyR2 are also controlled luminally through interactions with calsequestrin, junctin, and triadin [54]. RyR3 was found to co-localize with lysosomes in the perinuclear region of cardiac muscle cells, whereas RyR1 and RyR2 also co-localize with lysosomes they are 2-fold lower and mainly in the sub-plasmalemmal and extra-perinuclear, respectively, where 4- and 60-fold less lysosomal co-localization occurs [55].

### 3.2. The Inositol 1,4,5-Trisphosphate Receptor

The IP_3_R is the major Ca^2+^ release channel on the ER membrane of non-excitable cells [56]. Its presence in excitable cells serves to amplify the Ca^2+^ signal generated from depolarization [57]. The IP_3_R is also present in cardiac and skeletal muscle cells where it functions to increase gene expression through excitation-transcription coupling for fiber growth and stabilization of the neuromuscular junction [58,59]. The IP_3_R is a member of a vast ion channel superfamily [60]. Like RyR, the IP_3_R functions as a tetramer having a large cytosolic domain resembling a mushroom and six transmembrane segments with the Ca^2+^ binding portion homologous to that of the RyR [61]. Three isoforms of the IP_3_R exist with IP_3_R1 being the most thoroughly studied [62]. IP_3_ is generated as a second messenger from the breakdown of phosphoinositol-4,5-bisphosphate (PIP_2_) by phospholipase C (PLC) through activation of G-protein coupled receptors (GPCRs) [56]. Channel opening is activated by both Ca^2+^ and IP_3_, where IP_3_ increases the response of the channel to Ca^2+^ giving it a bell-shaped response curve similar to RyR [4]. Channel opening allows the release of Ca^2+^ from the ER and other internal stores expressing the receptor [56]. Several molecules interact indirectly or directly with the IP_3_R triggering its activation. ATP increases IP_3_-mediated Ca^2+^ release through the IP_3_R at concentrations of 100 µM [63]. ATP binds to the purinergic receptor P2YR, a GPCR, triggering production of IP_3_ that binds to the IP_3_R to release Ca^2+^ from the ER [64,65]. IP_3_R1 was also found to be activated by thiol modification with less than 10 µM of thimerosal, having more than this concentration actually caused an inhibitory effect [66]. Additionally, IP_3_R can be phosphorylated through protein kinase A (PKA), protein kinase B (PKB), cycline-dependent kinase 1 (CDK1), and MAP kinases [66].

### 3.3. The Sarco-Endoplasmic Reticulum Ca^2+^ ATPase Pump

The role of Ca^2+^ pumps is to direct Ca^2+^ ions out of the cell or back into organelles once the initial stimulus for the signaling event is removed. The major Ca^2+^ pump for the ER/SR is the SERCA pump found on the membrane. The SERCA pump is powered by ATP hydrolysis to move Ca^2+^ ions against the concentration gradient. This pump helps to restore cytosolic Ca^2+^ concentration to 0.1 µM [3,38]. The SERCA pump is a P-type ATPase pump that reduces cytosolic Ca^2+^ and refills the ER/SR by actively pumping the ion into the ER/SR lumen [41]. The pump functions as a monomer with a mass of 110 kDa. Three genes encode SERCA1, SERCA2, and SERCA3 isoforms whose amino acid sequences are highly conserved. From these three genes, alternative splicing generates over 10 variants of the pump adding to the diversity of its function in different tissues [67]. SERCA1 is primarily distributed in fast twitch skeletal muscle. SERCA2 is found in fast twitch and slow twitch muscle, cardiac muscle, smooth muscle, and non-muscle cells. SERCA3 is expressed in non-muscle cells [68]. The crystal structure of rabbit SERCA1a revealed a large cytosolic portion consisting of the Asp 351 phosphorylation site (domain P), the nucleotide-binding site (domain N), and the anchor portion (domain A). The transmembrane (domain M) portion of the pump contains 10 α-helices where two Ca^2+^ ions bind [69]. The binding sites are side by side and are formed by M4, M5, M6, and M8 helices. When Ca^2+^ binds to the pump, the large cytosolic head changes from a closed to open conformation to facilitate translocation of the ions. For each ATP molecule consumed, the pump transports two Ca^2+^ ions into the ER/SR lumen [68,70]. Specific inhibitors of the SERCA pump include thapsigargin, from *Thapsia garganica*, and cyclopiazonic acid (CPA), from *Aspergillus* and *Penicillum*. CPA has a low affinity for the pump and reversibly blocks the Ca^2+^ access point [71]. Thapsigargin, on the other hand, irreversibly binds to the Ca^2+^ free pump at residue F256 in the M3 helix with nanomolar affinity [41].

## 4. Local Domains of ER/SR Calcium Release

Intracellular calcium signaling is composed of both local and global events. Ca^2+^ channel activation produces momentary localized elevations in cytosolic Ca^2+^. These short-lived plumes, puffs, and sparks of Ca^2+^ are limited to 1–6 μm around the mouth of the channel. The diffusion of these brief bursts of Ca^2+^ sensitize nearby channels which aids signal amplification [72]. The recruitment and coordinated release of calcium from several calcium channels define global calcium signals. In skeletal muscle, synchronous channel opening is achieved through membrane depolarization that leads to the conformational change in the DHPR sensed by the coupled RyRs. The initial fast calcium release is via the DHPR coupled with RyR1. For cardiac muscle cells, calcium sparks facilitate CICR activating surrounding uncoupled RyRs to propagate the calcium signal [72]. The IP_3_R is also present in cardiac and skeletal muscle with IP_3_-associated calcium transients contributing to skeletal muscle growth and stabilization of the neuromuscular junction through excitation-transcription coupling, as mentioned in a previous section [58,59]. In non-excitable cells, Ca^2+^ puffs are responsible for producing regenerative Ca^2+^ waves and oscillations from the IP_3_R using CICR to recruit nearby clusters of IP_3_R when IP_3_ is present [10,73]. The structural framework and heterogeneous distribution of the ER/SR and associated proteins form localized Ca^2+^ signaling domains for the rapid mobilization of intracellular Ca^2+^ [21].

### 4.1. ER-PM Junction of Skeletal Muscle and Cardiac Cells

**Figure 2:**
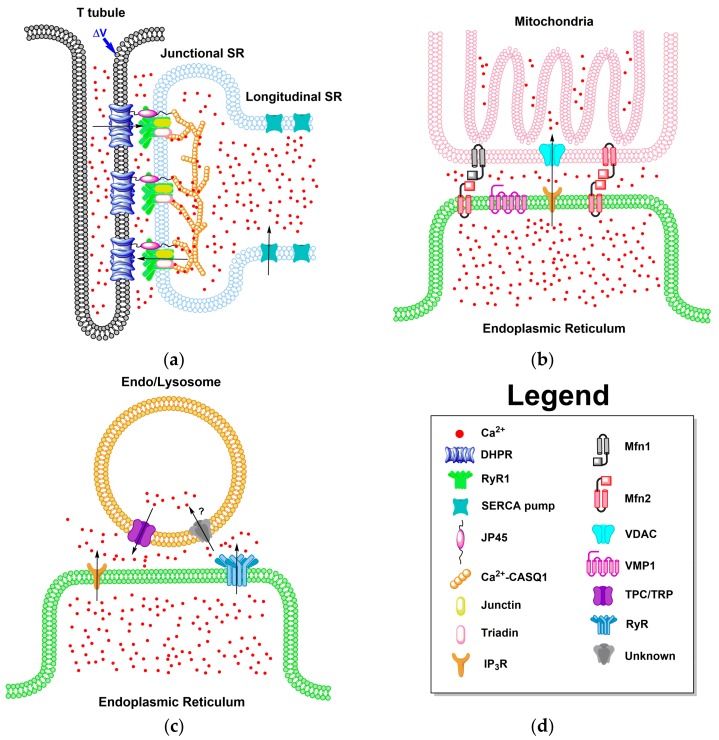
Membrane contact sites within the ER/SR. The black arrows indicate the direction Ca^2+^ flows through the respective receptor or pump. (**a**) Representation of the organization of the junctional zone and the channels, receptors, pumps, and proteins involved in the E-C coupling process in skeletal muscle cells. The Δ*V* here represents a voltage change applied to the cell membrane; (**b**) Representation of the mitochondria and ER/SR microdomain found in excitable and non-excitable cells; (**c**) Representation of the endo/lysosome and ER/SR microdomain found in excitable and non-excitable cells. The receptor/mechanism that returns Ca^2+^ to the endo/lysosome is indicated here by a “?” symbol, as it is still unknown; (**d**) Legend of symbols used in (**a**–**c**). DHPR (dihydropyridine receptor), JP45 (junctional protein 45), CASQ1 (calsequestrin), Mfn1 and Mfn2 (mitofusion proteins 1 and 2), VDAC (voltage-dependent anion channel), VMP1 (vacuole membrane protein 1), TPC (two-pore channels), and TRP (transient receptor channels).

As discussed in previous sections, the junctional zone is a highly specialized ER-PM junction found in skeletal muscle and cardiac cells. The structure of this interface with associated proteins allows an immense amount of Ca^2+^ to be released from the SR in response to action potentials, fueling E-C coupling [29,36,54] This large reservoir of Ca^2+^ required to generate contractile force repetitively from a train of action potentials in skeletal muscle cells is maintained by the low affinity, high capacity Ca^2+^ binding and release of calsequestrin (CASQ1) [74]. CASQ1 is concentrated in the SR TC where it forms long polymers near the opening of RyR1 in a Ca^2+^ dependent manner [75,76]. Polymerized CASQ1 can bind 40–50 mol of Ca^2+^/mol of CASQ1 with a 10^3^ M^−1^ affinity over a high Ca^2+^ concentration range of 0.01–1 M. Its exceptional buffering function makes CASQ1 the sole mechanism for fast Ca^2+^ binding and release from the SR [74]. CASQ1 is an important regulator of SR Ca^2+^ release through its association with integral membrane RyR associated proteins junctin and triadin (Figure 2a), but it is not required for luminal Ca^2+^ sensing. Under basal conditions, SR Ca^2+^ release and contraction were observed in studies of CASQ2-null mice [77]. When junctin and triadin are present, CASQ inhibits RyR function when luminal Ca^2+^ is at 1 mM. [78]. Research done by Manno and colleagues shows that CASQ polymerizes in the SR of adult mouse myofibrils at rest and depolymerizes fully in response to electrical stimulation or maximal depletion of Ca^2+^ from the SR by treatment with a high amount of 4-CmC supporting the luminal Ca^2+^ sensing role of CASQ [79]. In skeletal muscle cells, an additional protein associated with the junctional zone, having an important role in E-C coupling, was discovered called junctional protein 45 (JP45) [80]. JP45 interacts with the DHPR at the cytosolic portion of its N-terminal and with CASQ1 at its C-terminal situated in the SR lumen [81]. Deletion studies of JP45 from young mice resulted in a loss of skeletal muscle strength due to decreased expression of the DHPR, which is essential for E-C coupling [82]. These local Ca^2+^ signals that activate the contractile proteins in the junctional zone are fueled by global changes in SR Ca^2+^ and are critical for E-C coupling to occur [83]. The structure of this junction differs in cardiac cells. In heart cells, contraction is produced by calcium release from dyads composed of an adjacent TC and t tubule segment. The DHPR and the RyR isoform 2 (RyR2) are located in the dyad on the surfaces of the t tubule and SR membrane, respectively. Junctin, triadin, and CASQ all co-localize with the RyR2 as seen in skeletal muscle [84]. A lower density of DHPRs exists in cardiac cells. The DHPRs do not interact directly with RyR2 in response to depolarization. Instead, a steady current of extracellular Ca^2+^ comes in and activates RyR2 [85]. The Ca^2+^ sensor GCaMP6f, fused to the N-terminal of junctin and triadin, has been applied to study calcium release in the dyads of rat cardiomyocytes. Confocal imaging with the probe was able to elucidate Ca^2+^ nanosparks that are much smaller than Ca^2+^ sparks [86]. GCaMP2.2 and GCaMP2.2low were applied to study local Ca^2+^ release in the cleft region between the sarcolemma and junctional SR membrane in cardiac cells. Fusing the indicators with FKBP12.6 tagged them to the RyR microdomains. Using the targeted and non-targeted versions of the probe, cleft [Ca^2+^] was larger than the global, or bulk, [Ca^2+^] (194 nmol/L versus 100 nmol/L) [87].

### 4.2. ER Junctions with Other Organelles

The ER also has local junctions with other organelles such as mitochondria, lysosomes, and endosomes (Figure 2). The methods of communication between these organelles are being consistently researched and new mechanisms of interaction are elucidated frequently. The mitochondria and ER communication is as complex as it is vital. The IP_3_R on the ER releases Ca^2+^ from internal stores. If the mitochondria are within the critical distance, Ca^2+^ is taken into the mitochondria through the voltage-dependent anion channel (VDAC) and then into the cristae by the mitochondrial Ca^2+^ uniporter (MCU) [88,89]. The closer the distance between IP_3_R and MCU, the more Ca^2+^ is taken into the mitochondria from the ER/SR. Additionally, the further apart the two channels are, the less Ca^2+^ is shuttled between the organelles [90]. This critical distance, as well as ER structure, is shown to be regulated by the vacuole membrane protein 1 (VMP1), an ER transmembrane protein which assists in keeping the distance between mitochondria and the ER MCS [91]. The ER-mitochondria complex is further stabilized by the connection of mitofusion proteins, Mfn1 and Mfn2. The ER has been shown to have this transmembrane protein Mfn2 on the membrane forming a complex with either Mfn1 or Mfn2, both expressed on the mitochondrial membrane [92,93].

The MCS between the ER/SR and the endosomal and lysosomal pathway is as complex as it is biologically relevant. The Ca^2+^ signaling between these organelles is less readily understood, while being expanded upon through vigorous research daily. While studies have shown that the endo/lysosome contact with the ER/SR is extremely complex and intricate, the Ca^2+^ signaling is marginally more simplistic yet mysterious. In the MCS between the ER/SR and endo/lysosome, as shown in Figure 2c, IP_3_Rs and RyRs release Ca^2+^ from internal ER/SR stores, meanwhile the transient receptor channels (TRP) and two-pore channels (TPC) release Ca^2+^ from the endo/lysosome after activation by Ca^2+^ activator nicotinic acid adenine dinucleotide phosphate (NAADP) [94,95,96,97,98]. While the shutting of Ca^2+^ from the internal stores of ER/SR, as well as endo/lysosomes, the mechanism for pumping Ca^2+^ into the endo/lysosome is still unknown. Research shows that Ca^2+^ is brought into endo/lysosomes, but the protein which regulates this process and its functions is still being investigated, although based on the rates of uptake it seems a Ca^2+^ exchanger is more likely than a Ca^2+^ pump [99].

## 5. Diseases Associated with ER/SR Calcium Signaling

As cells grow and separate into their respective tissue types during the developmental process, diverse elements of the Ca^2+^ signaling machinery will be expressed that will render different properties to the generated Ca^2+^ signal [7,38]. These components are under continuous modification to adjust to environmental changes and ensure the preservation of the Ca^2+^-mediated response for the particular cell type. When problems arise with constituents of the signaling network, Ca^2+^ itself will trigger transcription of the defunct components to restore the signal [38]. Various diseases are attributed to dysfunctional elements in the Ca^2+^ signaling network [100,101], primarily the IP_3_R and RyR that mediate Ca^2+^ release from the ER/SR and the SERCA pump [16]. A few of the diseases and conditions that involve these major Ca^2+^ signaling elements will be discussed in this section.

### 5.1. Brody’s Disease

Brody’s disease is an uncommon genetic disorder of skeletal muscle [41]. The symptoms of this musculo-skeletal disease are not exact but are commonly described as cramps, reduced muscle relaxation, and exercise-induced stiffness. Since the symptoms are broad, diagnosing this disease in a clinical setting is problematic. Brody’s disease originates from mutations to SERCA1 through the ATP2A1 gene. The function of the pump, not its expression level, is decreased in suffering patients [18,102]. The SERCA pump is responsible for sequestering cytosolic Ca^2+^ into the ER/SR after the occurrence of the signaling event [103]. As a result of pump malfunction, cytosolic [Ca^2+^] remains high after stimulus removal. This sustained level of cytosolic Ca^2+^ contributes directly to muscle stiffness. Administration of the drugs dantrolene and verapamil was shown to restore cytosolic Ca^2+^ levels back to basal [102].

### 5.2. Catecholaminergic Polymorphic Ventricular Tachycardia

Ca^2+^ is a critical component of the various aspects of the signaling system in cardiomyocytes that work together to generate the contractile force needed to pump blood throughout the body [104]. Some of these areas include the electrophysiological mechanisms used to generate the action potential, E-C coupling, myofilament activation, energy manufacture and metabolism, cell death, and transcriptional control of cardiac machinery [105]. A breakdown or mutation in any of the components in the previously mentioned areas will cause electrical and mechanical problems within the heart [104]. Catecholaminergic polymorphic ventricular tachycardia (CPVT) is a heart disorder that causes abnormal heart rhythm [100]. CPVT is characterized by rapid heart rate through β-adrenergic receptor stimulation. Commonly occurring in kids and teenagers, CPVT causes fainting and sudden death. When left untreated, CPVT has a 31% mortality rate in patients 30 years of age [106]. The clinical characterization of CPVT is fainting connected to seizures brought on by stress or exercise. The primary form of CPVT, CPVT1, is caused by dominant mutations in RyR2. Mutations in CASQ2 also cause CPVT [100,107]. RyR2 plays a major role in Ca^2+^ regulation and release from the SR in cardiomyocytes [108]. Activation of the L-type Ca^2+^ channel allows Ca^2+^ to flow into the cytosol from the extracellular environment. The circulating Ca^2+^ opens RyR2, through CICR, allowing prompt discharge of Ca^2+^ from the SR. The elevated cytosolic Ca^2+^ level induces contraction and is taken back up into the SR [104,109]. Improper regulation of this cycle is associated with many heart disorders such as CPVT [106].

### 5.3. Malignant Hyperthermia

Malignant hyperthermia (MH) is a genetic skeletal muscle condition caused by mutations in RyR1 [110,111]. Commonly induced by certain anesthetics, MH is the underlying cause of anesthesia-related deaths in patients who are seemingly healthy. The anesthetics known to initiate MH include isoflurane, sevoflurane, haloethane, desflurane, enflurane, and the muscle relaxer succinylcholine [112]. In some reported cases of MH, abrupt changes in temperature and stressors triggered the disease. Clinical hallmarks of MH include rapid heart rate, high blood pressure, sustained muscle contractions, increased CO_2_ levels, trouble breathing, and severe increase in core temperature. Untimely diagnosis of MH leads to death. The hypermetabolic state mirrored in MH is attributed to escalated Ca^2+^ release from the SR caused by defects in RyR1 situated on the SR membrane [111,112]. Hundreds of mutations to human RyR1 have been linked to MH, thus far, with research still ongoing. The majority of these mutations are confined to three locales in RyR1: C35-R614 in the N-Terminal, D2129-R2458 in the central region, and I3916-G4942 in the carboxy terminal, but several mutations still exist outside of these areas [113]. MH mutations have also been identified in the human CACNL1A3 gene coding the α_1_ subunit of the DHPR located on the sarcolemma [114]. Different mechanisms have been revealed for the molecular basis of this condition. Dirksen and Avila found that RyR1 mutations, that only produce MH, slightly increased RyR1 activity without changing the overall concentration of SR Ca^2+^, whereas those that produce MH and central core disease (CCD) increase RyR1 activity resulting in depletion of the SR and an increase in intracellular Ca^2+^ [115]. Other studies reveal that MH mutations to RyR1 and CASQ1 decrease the threshold for store overload induced calcium release (SOICR). Exposure to volatile anesthetics further decreases the SOICR barrier, making RyR1 more sensitive to small increases in luminal Ca^2+^. CASQ1 MH mutants exhibit reduced buffering ability causing SR Ca^2+^ to increase over the threshold. This reduced threshold for Ca^2+^ activation of RyR1 leads to an increase in cytosolic Ca^2+^, causing the characteristic hypermetabolic state depicted in MH patients [116]. The abnormal handling of SR Ca^2+^ leads to muscle rigidity caused by elevated myoplasmic Ca^2+^ and increased glycogen and glucose breakdown sparked by phosphorylase kinase galvanization by Ca^2+^ [113]. These frenzied metabolic reactions consume O_2_, ATP, and glycogen reserves and create exorbitant amounts of metabolic waste products, leading to an eventual disturbance in ion levels and ensuing cellular destruction [117]. Clinical treatment of MH involves the administration of the muscle relaxer dantrolene, the only pharmacological agent known to treat the disease [118]. The mechanism for the action of dantrolene has been widely debated. Dantrolene imposes a hindrance on the DHPR, blocking L-type currents and diminishing Ca^2+^ release from the SR; however, expression of RyR1 was found necessary for dantrolene to exert an inhibitory effect on the DHPR through modification of the coupling between these channels [119]. Recently, dantrolene was shown to inhibit RyR1 and RyR2 function in the presence of 100 nM CaM in single channel recordings [120].

### 5.4. Alzheimer’s Disease

Alzheimer’s disease (AD) is medically defined as the continuous deterioration of mental capacity leading to noticeable declines in behavior and memory associated with aging [121]. Currently, the prevalence of AD is ranked highest among all other neurological maladies and has no cure. The majority of AD cases that arise are late onset or sporadic AD (SAD). Familial AD (FAD), or early onset AD, constitutes a small percentage of cases and has genetic origins. In both cases, AD manifests in the brain as lesions of amyloid-beta (Aβ) protein, fibrous tangles of tau (τ) protein, and cell death resulting in the reduction of brain mass and ultimately death [122,123]. Amyloid plaque formation is caused by the improper cleavage of the neuroprotective amyloid precursor protein (APP) by secretase enzymes resulting in copious amounts of the cytotoxic, 42 residue fragment Aβ [124]. Mutations in APP and presenilins 1 and 2 (PS1 and PS2), which are located in the ER membrane of neurons, lead to a buildup of Aβ in cases of FAD. A common denominator in all cases of AD is the mishandling of neuronal intracellular Ca^2+^, primarily IP_3_R and RyR-mediated Ca^2+^ release from the ER [121,125]. Resting free intracellular Ca^2+^ levels were dramatically increased in the neurons of transgenic mice exhibiting AD compared to normal neurons. This increase was due to the influx of Ca^2+^ through voltage-gated channels and its release from the ER through the IP_3_R and RyR [126]. PS1 and PS2 mutants of FAD were shown to excite IP_3_R activity causing amplified Ca^2+^ signaling from the ER, which increased Aβ production [127]. Injection of Aβ aggregates into Xenopus oocytes alone elicited IP_3_R stimulation through GPCR production of IP_3_ resulting in vigorous Ca^2+^ release from the ER and cytotoxicity [128]. Reduction in IP_3_R levels and blocking of RyR activity was shown to alleviate AD symptoms in AD mouse models solidifying IP_3_R and RyR-mediated Ca^2+^ release from the ER in neurons as therapeutic targets for AD [129,130].

## 6. ER/SR-Targeted GECIs

To decipher the irregularities in Ca^2+^ signaling that lead to the aforementioned diseases, specific tools are essential to study them. The progress made in the creation of synthetic calcium dyes yielded significant advances in the understanding of intracellular calcium signaling [131]. MagFura-2 and Fluo-5N are frequently used to study ER Ca^2+^, having dissociation constants (*K*_d_) of 25 and 90 µM, respectively [132,133,134,135]. The application of synthetic indicators for studying Ca^2+^ changes in specific organelles is plagued with the compartmentalization propensity of the dye in non-specific organelles [131]. The ability to monitor calcium transients, in situ and in vivo, in targeted intracellular locations was revolutionized by the discovery and manipulation of fluorescent proteins [136]. Using site-directed mutagenesis, circular permutation, and fused FP constructs, a variety of GECIs have been created to monitor cytosolic and organellar Ca^2+^, becoming powerful tools in the field of calcium imaging [137,138]. To measure luminal Ca^2+^ within a specific organelle like the ER/SR, there are preferable properties the indicator should have. First, the GECI must have specificity for Ca^2+^, over other physiological ions and small molecules, with a *K*_d_ between 0.5–5× the [Ca^2+^] of the target organelle. It must have good folding and bright fluorescence at 37 °C for mammalian cell application. It should possess a large signal enhancement upon Ca^2+^ binding with a high signal to noise ratio (SNR). It should display rapid binding and release kinetics for measuring fast calcium fluctuations. The binding stoichiometry of the GECI–Ca^2+^ complex should be 1:1 for simple, quantitative interpretation of the fluorescence signal. Additionally, the probe should be unaffected by the pH of the environment being monitored [137,138,139]. Here, we give a brief history on GECIs with a primary focus on ER/SR-targeted GECIs. For further reading, a few in depth reviews on calcium dyes and GECIs are referenced here [134,137,138].

GECIs fall into two major categories: single fluorophore and Förster resonance energy transfer (FRET) pair. Single fluorophore sensors are non-ratiometric and experience a change in fluorescence intensity when calcium binds. Most of the single fluorophore sensors are large chimeras of a fluorescent protein (FP) connected to a native CaBP, such as CaM or troponin C (TnC). Chelation of calcium by the CaBP induces a conformational change that rearranges the chromophore environment, inducing fluorescence change. For single fluorophore sensors, calcium binding produces changes either in absorbance or the quantum yield that lead to the change in fluorescence intensity. Single fluorophore sensors are commonly referred to as intensiometric because the fluorescence intensity change at only one wavelength is measured [137]. FRET pair, or ratiometric, sensors involve the fusion of two FPs with a CaBP in the center of the construct. In these indicators, the emission energy of one FP (donor) is used as the excitation energy for the other FP (acceptor). The efficiency of energy transfer is dependent on the distance between the two FPs and the extent of overlap between the emission and excitation of the donor and acceptor, respectively. The conformational change that occurs between the CaBP when calcium binds brings the FPs closer together for FRET to occur [137,140]. Cameleons are genetically encoded FRET pair sensors consisting of two fluorescent proteins, normally cyan fluorescent protein (CFP) and yellow fluorescent protein (YFP), with calmodulin and M13, from myosin light chain kinase, in the center of the construct. These sensors have been applied in different cell lines to monitor Ca^2+^ dynamics from the ER/SR and cytosol [141] but primary application is cytosolic due to their strong affinity for Ca^2+^. D1ER and D4cpv-CASQ1 are cameleons that utilize CFP and Citrine, a type of YFP, and CFP and circularly permutated Venus, respectively. D1ER and D4cpv-CASQ1 are targeted to the ER/SR and have been used to study Ca^2+^ release from these organelles [142,143]. Recently, ER-targeted cameleons with a red hue were developed based off of the optimal cameleon D1ER [142]. The new red shifted cameleon called D1ERCmR2 has the fluorescent proteins Clover positioned at the N-terminal acting as the FRET pair donor and mRuby2 at the C-terminal acting as the FRET pair acceptor. D1ERCmR2 has two in vitro *K*_d_s of 0.8 and 60 µM with an in situ *K*_d_ of 220 µM determined in HeLa cells. D1ERCmR2 can be used in tandem with fura-2 to measure ER and cytosolic Ca^2+^ concentrations [144]. As for the kinetics of cameleon sensors, not much data has been reported. Cameleons YC2.0, one of the first yellow cameleons, has a dissociation time constant (*Ƭ*_rt_) of 83 ms. YC-Nano140 has a *Ƭ*_rt_ of 303 ms [137].

Because of the large size of FRET pairs and limited signal intensity, probes called GCaMP were developed using a single circularly permuted enhanced green fluorescent protein (cpEGFP) fused to calmodulin and M13. The use of cpEGFP increased the signal intensity of the probes for use in cells but their affinities for Ca^2+^ are high with *K*_d_ values in the nanomolar range [145,146]. The original GCaMP indicator, published in 2001, has a Ca^2+^ *K*_d_ of 235 nM with a dissociation time constant of ~200 ms [145]. The Ca^2+^ induced fluorescence change mechanism was discovered from crystal structure analysis of GCaMP2. The Ca^2+^ induced conformational change in CaM creates new contacts between CaM and cpGFP. CaM residues change the chromophore environment of cpGFP, preventing solvent access to the chromophore aiding fluorescence increase [146]. Since its unveiling, several variants of GCaMP have been created and used to monitor Ca^2+^ transients in different environments such as mouse cardiac cells in vivo (GCaMP2) [147], chemosensory neurons of *Caenorhabditis elegans* and *Drosophila melanogaster* (GCaMP3) [148], and in the brain cells of mice, *Caenorhabditis elegans*, *Drosophila melanogaster*, and zebra fish (GCaMP5s) [149]. As mentioned in a previous section, GCaMP6f is a newer construct in the GCaMP family with the ability to sense Ca^2+^ nanosparks that initiate EC coupling in the junctional zone dyad space in rat cardiomyocytes. By targeting the sensor to the junctional zone using resident proteins junctin and triadin, Ca^2+^ nanosparks that are 50 times smaller than standard sparks were seen [86]. All of the initial GCaMP variants mentioned have strong affinities for Ca^2+^ that limit their use to cellular environments with minimal Ca^2+^ concentrations like the cytosol, and only use cpGFP. None of the initial GCaMPs have Ca^2+^ affinities low enough to target to organelles like the ER/SR [145,147,149]. A new subfamily of GCaMPs was created, from random mutations to GCaMP3, termed genetically encoded Ca^2+^ indicators for optical imaging (GECOs). Green (G-GECO), red (R-GECO), and ratiometric blue-green (GEM-GECO) variants were created. Although the color palette was expanded, the new GECO variants still have high Ca^2+^ sensitivity [150]. Robert Campbell and colleagues created low affinity red (LAR) GECOs, LAR-GECO1 and LAR-GECO1.2, with Ca^2+^ *K*_d_s of 24 and 12 µM, respectively. LAR-GECO1 was used to monitor thapsigargin inhibition of ER refilling in HeLa, HEK293, and U2-OS cells co-transfected with CatchER. LAR-GECO had larger decreases in intensity over all three cell lines [151]. In 2014, new low affinity, GECO-type indicators were created based on cfGCaMP2. The lead variant, from a library of 58 mutants, had a Ca^2+^ *K*_d_ of 368 µM and a large dynamic range. This new variant termed Ca^2+^-measuring organelle-Entrapped Protein IndicAtor 1 in the ER (CEPIA1er) was able to monitor ER Ca^2+^ dynamics in HeLa cells with thapsigargin and histamine treatment [152]. Variants of CEPIA were also created with different emission wavelengths such as red (R-CEPIAer), green (G-CEPIAer), and ratiometric blue-green (GEM-CEPIAer) with Ca^2+^ affinities of 565, 672, and 558 µM, respectively [152]. Table 1 lists properties of the discussed GECIs. Many of the mentioned and reported GECIs, such as CEPIA, do not state their Ca^2+^ binding and release kinetics. For more localized measurements of rapid Ca^2+^ transients, in processes like muscle contraction, it is crucial to utilize a Ca^2+^ biosensor with reported, fast kinetics [153].

**Figure 3:**
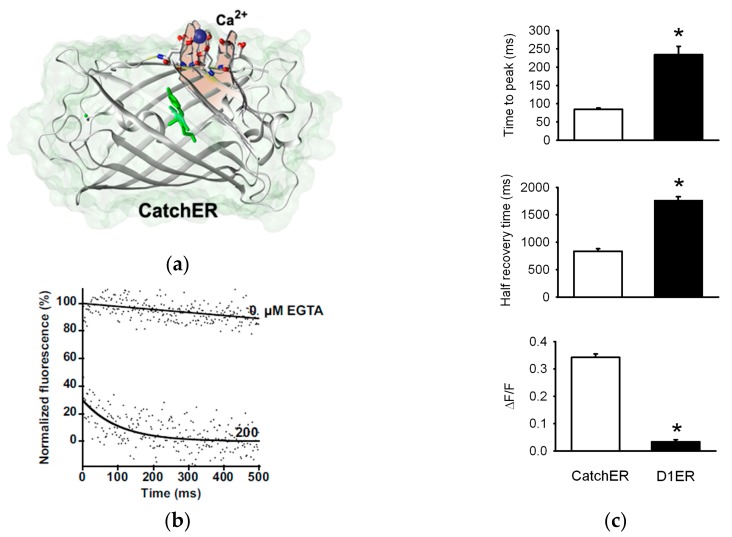
Capturing fast Ca^2+^ dynamics with CatchER. (**a**) Cartoon representation of the crystal structure of Ca^2+^ bound CatchER (PDB: 4l1i). Using site-directed mutagenesis, a Ca^2+^ binding site consisting of mutated residues S147E, S202D, Q204E, F223E and T225E was created on the surface of enhanced green fluorescent protein (EGFP); (**b**) *k*_off_ traces measured by stopped-flow. Fluorescence decreased when Ca^2+^ bound CatchER was mixed with 200 µM ethylene glycol-bis(β-aminoethyl ether)-*N*,*N*,*N*′,*N*′-tetraacetic acid (EGTA). Measurements were carried out in 10 mM Tris (pH 7.4) at room temperature; (**c**) CatchER and D1ER kinetics in response to 100-ms pulses to 20 mV in FDB fibers under patch clamp. Time to peak, half recovery time, and response range normalized to basal fluorescence (Δ*F*/*F*) were analyzed for both indicators. (* *p* < 0.01).

In the past, we demonstrated our knowledge and skill for designing novel CaBPs using non-native CaBPs as the scaffold [154,155], and manipulating the binding affinity of these designed CaBPs by altering the number of charged residues in the metal binding site [156,157]. Using these previously published results and subsequent statistical analysis of coordination chemistry in native CaBPs [158,159], a novel EGFP-based sensor called CatchER (Ca^2+^ sensor for detecting high concentration in the ER) was designed [160] (Figure 3). CatchER has a similar absorption profile as GFP with 395 nm and 488 nm excitation peaks and single wavelength emission at 510 nm. CatchER has a large fluorescence intensity change induced by Ca^2+^ binding and a *K*_d_ between 120–180 µM. Its unmatched Ca^2+^ off rate (*k*_off_) of 700 s^−1^ (Figure 3b), which is unique for a GECI, makes it capable of measuring fast Ca^2+^ release from the ER/SR in various cell types [160]. In response to electrical stimulation of flexor digitorum brevis (FDB) muscle fibers electroporated with CatchER, the probe had a faster response time and larger dynamic range compared to cells electroporated with D1ER [160] (Figure 3c). Although CatchER demonstrates 1:1 binding stoichiometry for Ca^2+^, crystal structure analysis revealed two positions for the Ca^2+^ ion bound within the designed site created by incorporating mutated residues S147E, S202D, Q204E, F223E and T225E. The multiple occupancies for a single Ca^2+^ ion, within the binding site, were deemed responsible for the rapid kinetic properties of the sensor [161]. CatchER also has a 44% in fluorescence lifetime with Ca^2+^ bound [162], a property not found in current ER/SR-targeted GECIs. This Ca^2+^-induced fluorescence lifetime increase points to its future application in fluorescence lifetime imaging microscopy (FLIM). FLIM provides better spatial resolution on smaller timescales extending its quantitative ability beyond standard fluorescence microscopy [163].

## 7. Summary and Perspective

The intricacies of the intracellular calcium signaling network are impacted by the structural organization of the ER/SR. Events such as muscle contraction are greatly influenced by the local organization of the RyR, CASQ, JP45, and other regulatory proteins that initiate Ca^2+^ release from the SR lumen. A better understanding of local Ca^2+^ signaling and the essential components that comprise the underlying machinery will guide the development of innovative methods to treat the numerous diseases that develop as a result of impaired ER/SR Ca^2+^ signaling.

## Figures and Tables

**Table 1:** Properties of ER/SR Ca^2+^ indicators.

Sensor Name	*k*_on_ (M^−1^·s^−1^)	*k*_off_ (s^−1^)	*K*_d_ (µM)	*λ*_Ex_ (nm)	*λ*_Em_ (nm)	Reference
CatchER	3.89 × 10^6^	700	120–180	395, 488	510	[160]
D1ER	3.86 × 10^6^	256	0.8–60	436 (CFP)	465 (CFP)	[164]
				500 (YFP)	535 (YFP)	
D1ERCmR2	-	-	200	490	510,560	[144]
Fluo-5N	-	-	90	494	516	[165]
G-CEPIA1er	-	-	672	(−Ca^2+^) 402,498	(−Ca^2+^) 499	[152]
				(+Ca^2+^) 401,497	(+Ca^2+^) 498	
GEM-CEPIA1er	-	-	558	(−Ca^2+^) 401	(−Ca^2+^) 381,395	[152]
				(+Ca^2+^) 391	(+Ca^2+^) 381,394	
LAR-GECO1	-	-	24	(−Ca^2+^) 574	598	[151]
				(+Ca^2+^) 561		
LAR-GECO1.2	-	-	12	(−Ca^2+^) 570	(−Ca^2+^) 594	[151]
				(+Ca^2+^) 557	(+Ca^2+^) 584	
Mag-Fura-2	7.5 × 10^8^	600, 26, 760	50	345	490	[135]
R-CEPIA1er	-	-	565	(−Ca^2+^) 445,576	(−Ca^2+^) 570	[152]
				(+Ca^2+^) 448,562	(+Ca^2+^) 561	

*k*_on_—Rate of calcium association to the indicator. Obtained using stopped-flow. *k*_off_—Rate of calcium dissociation from the indicator. Obtained using stopped flow. *K*_d_—dissociation constant. *λ*_Ex_—excitation wavelength(s) with (+Ca^2+^) and without (−Ca^2+^) calcium. *λ*_Em_—emission wavelength(s) with (+Ca^2+^) and without (−Ca^2+^) calcium.

## Abbreviations

Ca^2+^	Calcium
ER	Endoplasmic reticulum
SR	Sarcoplasmic reticulum
RER	Rough ER
SER	Smooth ER
CaBP	Calcium-binding proteins
GECI	Genetically encoded calcium indicators
IP_3_	Inositol 1,4,5-triphosphate
IP_3_R	Inositol 1,4,5-triphosphate receptor
RyR	Ryanodine receptor
cADPR	Cyclic ADP-ribose
CICR	Calcium induced calcium release
SERCA	Sarco-endoplasmic reticulum Ca^2+^ ATPase
UPR	Unfolded protein response
MCS	Membrane contact sites
PM	Plasma membrane
MAM	Mitochondria-associated ER membrane
NE	Nuclear envelope
TC	Terminal cisternae
CaM	Calmodulin
4-CmC	4-Chloro-m-cresol
E-C	Excitation-contraction
DHPR	Dihydropyridine receptor
PIP_2_	Phosphoinositol-4 5-bisphosphate
Mg^2+^	Magnesium
PLC	Phospholipase C
GPCR	G-protein coupled receptors
PKA	Protein kinase A
PKB	Protein kinase B
CDK1	Cycline-dependent kinase 1
CPA	Cyclopiazonic acid
JP45	Junctional protein 45
CASQ1	Calsequestrin (skeletal muscle)
VDAC	Voltage-dependent anion channel
MCU	Mitochondrial Ca^2+^ uniporter
VMP1	Vacuole membrane protein 1
TRP	Transient receptor channels
Mfn	Mitofusion proteins
TPC	Two-pore channels
NAADP	Nicotinic acid adenine dinucleotide phosphate
CPVT	Catecholaminergic polymorphic ventricular tachycardia
MH	Malignant hyperthermia
ATP	Adenosine triphosphate
AD	Alzheimer’s disease
SAD	Sporadic AD
FAD	Familial AD
Aβ	Amyloid-beta
τ	Tau
APP	Amyloid precursor protein
PS1	Presenilins 1
PS2	Presenilins 2
SNR	Signal to noise ratio
TnC	Troponin C
FP	Fluorescent protein
Ƭ_rt_	Dissociation time constant
cpEGFP	Single circularly permuted enhanced green fluorescent protein
FDB	Flexor digitorum brevis
FLIM	Fluorescence lifetime imaging microscopy
FRET	Förster resonance energy transfer
CFP	Cyan fluorescent protein
YFP	Yellow fluorescent protein
